# High-frequency sound components of high-resolution audio are not detected in auditory sensory memory

**DOI:** 10.1038/s41598-020-78889-9

**Published:** 2020-12-10

**Authors:** Hiroshi Nittono

**Affiliations:** Graduate School of Human Sciences, Osaka University, Suita, 565-0871 Japan

**Keywords:** Neuroscience, Psychology

## Abstract

High-resolution digital audio is believed to produce a better listening experience than the standard quality audio, such as compact disks (CDs) and digital versatile disks (DVDs). One common belief is that high-resolution digital audio is superior due to the higher frequency (> 22 kHz) of its sound components, a characteristic unique to this audio. This study examined whether sounds with high-frequency components were processed differently from similar sounds without these components in the auditory cortex. Mismatch negativity (MMN), an electrocortical index of auditory deviance detection in sensory memory, was recorded in young adults with normal hearing (*N* = 38) using two types of white noise bursts: original sound and digitally filtered sound from which high-frequency components were removed. The two sounds did not produce any MMN response and could not be discriminated behaviourally. In conclusion, even if high-resolution audio is superior to the standard format, the difference is apparently not detectable at the cortical level.

## Introduction

High-resolution audio is a collective term for digital audio formats and systems that can handle higher informational content than traditional digital audio, such as compact disks (CDs) and digital versatile disks (DVDs). The sampling rate and quantization depth of high-resolution audio exceed 44.1 or 48 kHz and 16 bits, respectively^1^. Since the late 1980s, high-resolution audio has undergone many technological advancements and is now gaining popularity rapidly due to the proliferation of electronically distributed content on the Internet (either downloads or streaming)^2^. Technically, it can record and render analog sound sources more accurately than the conventional format. However, whether high-resolution audio produces a better listening experience than standard quality audio is debatable, because the standard format also covers the full spectrum of the human audible range (ca. 20–20,000 Hz). In fact, it is not always easy to discriminate the two sound formats. A meta-analysis of 17 studies published between 1980 and 2016 showed that discrimination performance was only slightly better than chance (*M* = 52.3% with a 95% confidence interval of 50.6–54.0%)^3^. Participants who received detailed training and instructions tended to get a better score, but they never became perfect at the exercise.

The higher-than-standard sampling rate of high-resolution audio enables it to reproduce higher frequency sounds that exceed the human audible threshold, because a digital signal can express frequency components up to half its sampling rate. Oohashi and colleagues (2000) conducted a series of experiments and reported that listening to musical excerpts that were rich in high-frequency sound components (i.e. gamelan, an ensemble music in Indonesia which mainly consists of metal percussions) produced a higher alpha power (8–13 Hz) in the electroencephalogram (EEG) of the listener when the sound was presented through a full-range sound reproduction system than when the same sound was presented without high-frequency components^4^. They called this finding the “hypersonic effect”^4–8^. Mere presentation of high-frequency sounds did not produce any effects^4^. In addition, the comfortable listening level to which the listener adjusted the sound intensity was higher for full-range sounds than for high-cut sounds^5,6^. These effects peaked when the intensity of high-frequency components was increased by 6 dB^6^. Another research group has replicated the finding in another type of music (J. S. Bach’s cembalo music); listening to full-range presentations of musical excerpts rich in high-frequency components produced a greater EEG alpha power than listening to the same excerpts without high-frequency components^9,10^. The latter studies showed that the two excerpts with or without high-frequency components were not associated with reliable differences in subjective sound impressions or other psychophysiological measures, such as heart rate, heart rate variability, skin conductance level, and facial electromyograms over corrugator supercilii and zygomaticus major.

The mechanism of how inaudible sounds affect a listener’s EEG remains unclear. A study using magnetoencephalograms showed that a pure tone burst (70 ms in duration) did not evoke any noticeable brain responses for frequencies of 20,000 Hz and higher^11^. Oohashi et al. (2006) reported that high-frequency sound components did not affect EEG power when they were presented via earphones rather than loudspeakers^7^. Based on this finding, they proposed that some unknown information channel for vibration (either neuronal or non-neuronal), rather than the air-conducting auditory system, was responsible for the effect. Ultrasonic vibrations of 100 kHz or over applied directly to the skull (i.e. mastoid) are known to produce auditory sensation through bone conduction and resonance^12^. They are not felt as vibratory or thermal sensations, but rather as normal tones with a lower frequency^13^. Similar to auditory stimulation, they produce brain evoked responses^14^ and mismatch detection responses to frequency difference^15^ and stimulus omission^16^, suggesting that the common auditory pathway is used for bone-conducted ultrasound and air-conducted normal sound perception. This bone-conduction mechanism, however, does not fully account for the hypersonic effect, because high-frequency sound components are imperceptible when presented alone.

Another possible mechanism responsible for the differences between the full-range and high-cut sound materials is the distortion of acoustic information inherent in digital audio processing. The digital audio format expresses data discretely with sound components up to half the sampling rate (i.e. the Nyquist frequency). When reducing the sampling rate, an anti-alias filter is applied to remove high-frequency components that exceed the Nyquist frequency^17^. As a result, the auditory signal is also modulated in time. Specifically, when the signal contains an instantaneous rise or fall, the onset or offset is temporally blurred (called time smearing) and may influence auditory perception^18^.

In either case, the mechanism of how the brain detects sound differences remains unknown. Based on an experiment using positron emission tomography, Oohashi et al. (2000) suggested the role of subcortical structures in listening to sound materials rich in high-frequency components; the brain stem and thalamus were more activated to sounds with high-frequency components than those without them^4^. The difficulty in conscious perception^3,9,10^ also suggests that the high-frequency sound components are not detected in the cortex.

In this study, mismatch negativity (MMN)^19^, a commonly used index of auditory deviance detection in sensory memory measured as part of the EEG, was used to examine whether sounds with or without high-frequency components are processed differently at the cortical level. MMN is elicited by a perceptual mismatch from the preceding stimulus context in various dimensions, such as frequency, duration, intensity, and location^20^. To deal with a broad frequency range, white noise bursts containing all frequencies with a constant intensity were used as the eliciting stimuli^21^. Considering the two possible mechanisms mentioned above, the experiment was conducted using high-resolution grade loudspeakers and sounds smeared by anti-alias digital filters. A full-range white noise burst with a higher total sound energy was assigned to the standard stimulus, whereas a high-cut white noise burst with a lower energy was assigned to the deviant stimulus. This combination simplified the interpretation of the difference between the deviant and standard stimuli: any observed difference reflects the mismatch detection response in the auditory sensory memory, rather than an exogenous potential evoked by afferent stimulation^22^. If the cortex is capable of registering the characteristics of a high-resolution grade sound (i.e. rich in high-frequency components and/or sharp onset and offset), it is expected that infrequent occurrence of high-cut sounds in a sequence of full-range sounds would elicit MMN. For comparison, a similar test was conducted using a high-cut white noise burst from which not only inaudible but also audible high-frequency components were removed. An obvious MMN response was expected in the latter case. According to the standard procedure, MMN was recorded while participants watched a silent movie clip and did not pay attention to white noise bursts. After the EEG recording, a standard behavioural discrimination test, called the ABX test, was applied to assess the participants’ audibilities of the difference^3^. Both EEG and behavioural tests were conducted in a double-blind fashion.

## Results

**Figure 1:**
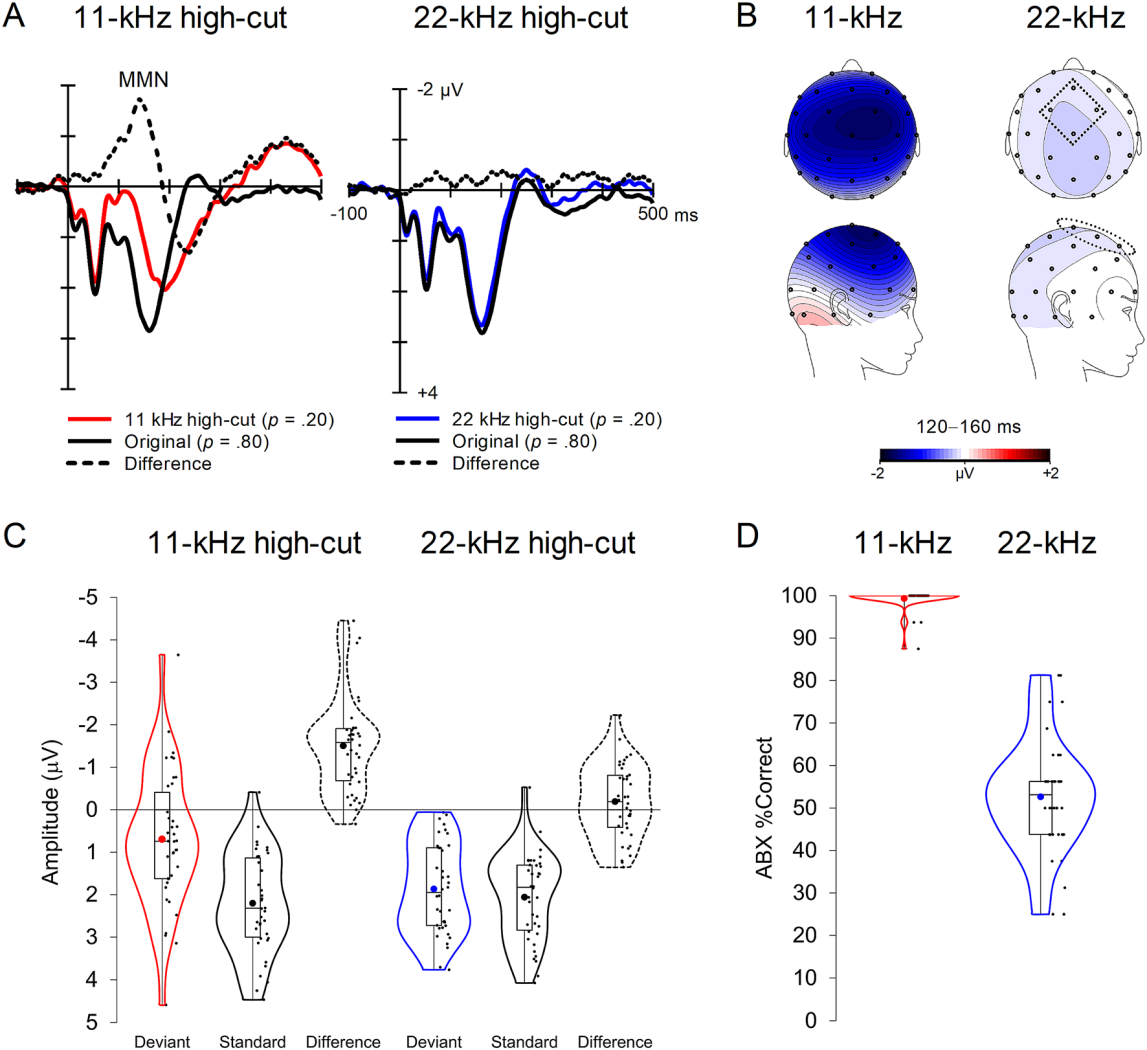
Results of mismatch negativity (MMN) response and behavioural discrimination. (**A**) Grand mean waveforms of the event-related brain potential to white noise bursts (*N* = 38). (**B**) Scalp topographic maps of the MMN response. (**C**) Amplitude of the MMN latency range (120–160 ms). (**D**) Accuracy of the ABX auditory discrimination test. In sum, a white noise burst produced an MMN response and could be discriminated behaviourally only when it lacked audible sound components.

All the participants reported no history of hearing problems. Their high-frequency auditory thresholds were between 14,000 and 19,000 Hz (*M* = 17,316 Hz). Figure 1A shows the grand mean event-related potential (ERP) waveforms of 38 university students. MMN occurred in response to the 11-kHz high-cut white noise bursts from which both audible and inaudible high-frequency components were removed, but not for the 22-kHz high-cut white noise bursts from which only inaudible high-frequency components were removed. Figure 1B,C show the scalp topography of MMN in the 120–160 ms period and its mean amplitudes over the frontocentral region (mean of Fz, FC1, FC2, and Cz). A Condition (11-kHz high-cut vs. 22-kHz high-cut) × Stimulus (deviant vs. standard) repeated measures analysis of variance revealed a significant interaction, *F*(1, 37) = 40.12, *p* < 0.001, η_p_^2^ = 0.52. Separate *t* tests showed that MMN was elicited by the 11-kHz high-cut sound, *t*(37) = 8.28, one-tailed *p* < 0.001, *d*z = 1.34, but not for the 22-kHz high-cut sound, *t*(37) = 1.34, one-tailed *p* = 0.094, *d*z = 0.22. For the latter, a Bayesian paired sample *t* test showed a Bayes factor of 0.718, which supported the null hypothesis (i.e. no MMN elicitation) over the alternative hypothesis (i.e. MMN elicitation). In contrast, the Bayes factor exceeded 3 × 10^7^ for the 11-kHz high-cut sound, which strongly supported the MMN elicitation. An exploratory analysis using all scalp electrodes to find difference potentials showed that the deviant and standard ERP waveforms did not differ at any time point for the 22-kHz high-cut sound, whereas significant differences were found in the 80–181, 201–285, and 418–500 ms periods for the 11-kHz high-cut sound (see Supplementary Figure S1).

Figure 1D shows the results of the ABX discrimination test. While the 11-kHz high-cut sound was distinguishable from the original sound (*M* = 99.3%, range = 88–100%), the 22-kHz high-cut sound could not be discriminated (*M* = 52.6%, range = 25–81%). According to the binominal distribution, 12 or more correct answers out of 16 trials (> 75%) were regarded as exceeding the chance level (*p* < 0.05, one-tailed). Four out of 38 participants (10.5%) exceeded the chance level in the 22 kHz high-cut sound condition. However, their mean MMN amplitude was − 0.07 μV, which was lower than the mean of all the participants (− 0.19 μV). On the whole, the correct detection rate was not significantly correlated with the MMN amplitude, *r*s(36) =  − 0.07 and 0.05 for the 11- and 22-kHz high-cut sound conditions, respectively, *p*s > 0.10, or with the auditory threshold, *r*s(36) = 0.25 and 0.13 for the 11- and 22-kHz high-cut sound conditions, respectively, *p*s > 0.10.

## Discussion

The present study showed that a sound from which inaudible high-frequency components were removed by digital filtering was not detected at the sensory cortical level or discriminated behaviourally. As loudspeakers (rather than earphones) were used, the high-frequency sound energy should have reached the listener’s body and brain through an unknown information channel for vibration, if it exsisted^7^. Moreover, the high-cut sounds examined in this study contained a temporal distortion (i.e. blurred onset and offset), which might be detected in the conventional auditory pathway. The lack of the corresponding electrocortical responses suggests that the auditory sensory memory cannot register the characteristics of a high-resolution grade sound (both high-frequency components and sharp onset and offset) and that the advantage of high-resolution audio, if any, should occur at the subcortical level and outside consciousness. This conclusion is further supported by the result that no ERP difference was found not only in the MMN latency range but also in the entire waveform.

In this study, the MMN response was used as an electrophysiological index of sensory memory and deviance detection at the cortical level. Another hypothesis suggests that MMN does not reflect a distinct functional unit called sensory memory but a habituation of afferent signal processing^23^. Even if this is true, MMN can be seen as an index expressing detection of a salient change in external stimulus features by the auditory system^24^. The current finding indicates that temporal smearing of instantaneous sound onsets induced by the anti-alias filter was not detected in the cortex.

In common speech, the merit of high-resolution audio is often referenced to its greater range of information. However, digital sound content is audible only after the audio is converted into analog signals. The whole process of the digital–analog (DAC) conversion to physical air vibrations by loudspeakers or headphones should be considered^18^. Spoiling the strength of high-resolution digital format by using a poor playback device and recovering the loss of the standard audio format by using a high-grade rendering system are both feasible. One potential merit of the high-resolution audio over the standard audio is its higher fidelity across different playback devices. Digital sound material needs to be interpolated between consecutive sampling points before it can be played back. The lower the sampling rate, the higher the susceptibility of the resulting analog signal to the DAC specifications of different playback systems. High-resolution audio formats are better than the conventional audio format, because the former exerts better control over the analog signal quality.

Strictly speaking, the present study did not address the discrimination between high-resolution audio and standard audio, because the equipment used in this study was high-resolution grade and both sound materials were created in a high-resolution format (192 kHz/24 bits), except that one of them did not contain high-frequency components. Moreover, the current finding does not deny the existence of audiophiles with the ability to discriminate between the original sound and filtered and blurred sound without high-frequency components. Nevertheless, at least for people with regular hearing ability, the broad playback bandwidth of high-resolution audio does not seem to have an advantage over the traditional standard audio’s bandwidth at a conscious level, although the former does no harm (except for the cost). Another characteristic feature of high-resolution audio, namely quantization depth or the precision of analog–digital conversion, was not manipulated in this study. The mechanism through which it affects listeners’ experiences is a topic of future research.

## Methods

This study was conducted using a preregistered design (https://osf.io/y5qfv/) and in a double-blind fashion. Both experimenters and participants did not know the contents of the audio files and experimental hypotheses. Several minor differences from the registered protocol occurred, but none of these were crucial. First, the criterion of ERP data exclusion was modified, because the reliabilities of the ERP waveforms were low (see below). Second, the delay between a stimulus trigger and a sound onset was recalibrated by an oscilloscope and set to 4 ms. Third, sound intensity was remeasured and determined as 62 dB (A).

Forty-four university students participated in the study. They gave written informed consent and received a cash voucher as an honorarium. Due to technical failure and low reliability of the ERP waveforms, the data of 6 participants were excluded, and the remaining data (*N* = 38, 18 men and 20 women, 19–23 years old, *M* = 21.6) were used for hypothesis testing. This sample size (36 or more) was determined to ensure the detection of the smallest effect size of interest (*d*z = 0.3) by a one-tailed *t* test (i.e. the difference amplitude between the deviant and standard stimuli was more negative than zero). This effect size was thought to be small enough, as 61.8% of the participants would have shown a difference in the expected direction. Alternatively, this sample size can detect an effect size of 0.50 with a power of 0.90, or an effect size of 0.56 with a power of 0.95 (see a previous study^25^ for the detailed calculation). The protocol was approved by the Behavioural Research Ethics Committee of the Osaka University School of Human Sciences, Japan. The experiment was performed in accordance with relevant regulations and guidelines, including the Code of Ethics and Conduct of the Japanese Psychological Association.

### Stimuli and apparatus

**Figure 2:**
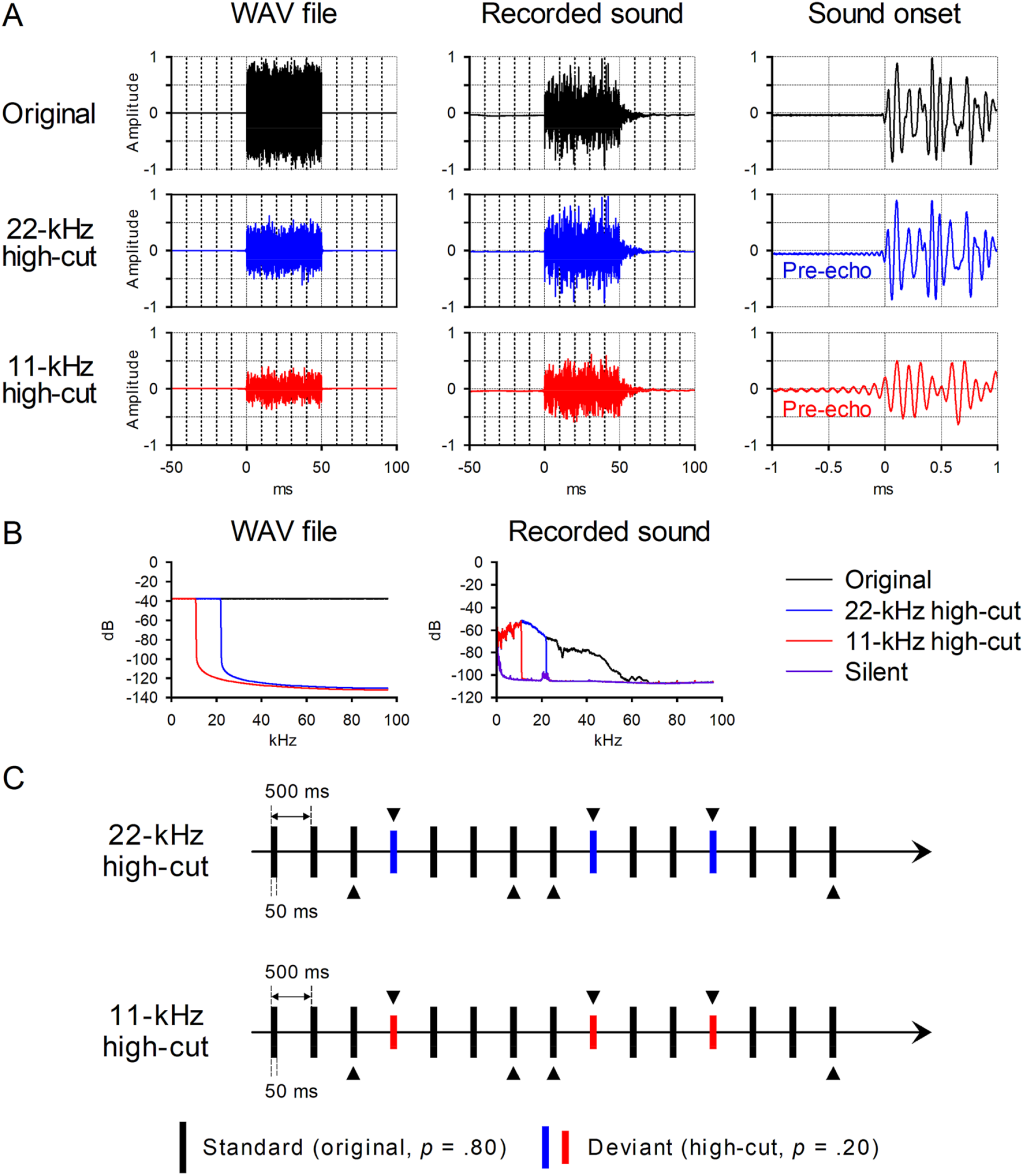
White noise burst stimuli used in the present study. (**A**) Digital data and recorded sound waveforms of the three types of white noise bursts. Amplitudes are shown in arbitrary units. The sounds were recorded by an electronic condenser microphone (Sony ECM-100U, Japan; frequency = 20–50,000 Hz) and an analog–digital converter (Roland Rubix24, Japan) with the 192-kHz/24-bit format. (**B**) Frequency characteristics of the sounds. The recorded sounds did not reproduce the digital data exactly because of ambient noise and the properties of playback and recording devices. Nevertheless, the three types of sounds differed distinctively from each other in terms of high-frequency components. (**C**) Sample of a stimulus sequence. At least two standard sounds were presented before a deviant sound. The solid triangles show the stimuli included in the analysis: standard and deviant sounds that were preceded by at least two standard sounds. An identical sequence was used in the 11- and 22-kHz high-cut sound conditions. The order of the conditions was counterbalanced across the participants.

Figure 2A,B illustrate the properties of auditory stimuli used in this study. A 50-ms white noise burst (192-kHz sampling rate, 24-bit resolution, and monaural) was created by Adobe Audition CC (Adobe Systems Incorporated, CA). Silent periods of 225 ms were added before and after the burst, creating a 500-ms WAV sound file (uncompressed PCM format). The sound was downsampled to either a 44,100- or a 22,050-Hz sampling rate, and upsampled again to the original 192-kHz sampling rate using a default sinc function of Adobe Audition. Undithered quantization was used to reduce the noise floor. The impulse responses of the anti-alias filters used for resampling are shown in Supplementary Figure S2. This procedure produced the two white noise bursts that did not contain high-frequency components over 22 or 11 kHz, respectively. For convenience, they are called 22-kHz high-cut sound and 11-kHz high-cut sound, respectively. In this study, the original noise burst was used as a standard (frequent) stimulus, whereas the 22- and 11-kHz high-cut sounds were used as a deviant (rare) stimulus in separate blocks. This was to ensure that the MMN response to deviant stimuli was elicited by perceptual mismatch in the auditory sensory memory, not by higher sound energy of a stimulus.

The WAV files (available at https://osf.io/y5qfv/) were presented by a software audio player (foobar2000, https://www.foobar2000.org/) on a Windows laptop PC. A USB digital–analog converter (Meridian Ultra, UK), a preamplifier (Yamaha A-U671, Japan), and two loudspeakers (Yamaha NS-BP401, Japan) were used. All the audio equipment was designed for high-resolution audio.

### Procedure

The MMN response was recorded with a standard protocol complying with the published guidelines^26^. Figure 2C shows a sample of a stimulus sequence. The stimuli were presented in a random order (standard *p* = 0.8 and deviant *p* = 0.2) under the constraint that at least two standards were presented before each deviant. The onset-to-onset interval of stimuli was 500 ms. A total of 1,000 stimuli (800 standards and 200 deviants) were presented for ERP recording in each of the 11- and 22-kHz high-cut sound conditions. The sounds were delivered through the two loudspeakers placed 1.2 m in front of the participants with an intensity of 62 dB (A). The delay between the onset of the stimulus trigger (StimTrak, Brain Products, Germany) and the sound onset at the ear level was 4 ms, which was measured using a microphone and an oscilloscope. The participants were told to watch a silent cartoon movie and ignore the sounds. The order of the 11- and 22-kHz high-cut sound conditions was counterbalanced across the participants. The two conditions were run without a break.

The EEGs from 34 scalp sites and the left and right mastoids were used for analysis, with the nose tip as the reference electrode. Horizontal and vertical electrooculograms were also recorded. The data were collected by QuickAmp (Brain Products, Germany) and analysed by Brain Vision Analyzer (Brain Products, Germany). The recording sampling rate was 1000 Hz. Online filters of DC–200 Hz and offline digital filters of 1–30 Hz were applied. Bad electrodes were interpolated. Ocular artifacts were corrected using the method proposed by Gratton et al.^27^.

To assess perceptual discrimination ability, two ABX tests were conducted by the ABX comparator component of the software audio player (https://www.foobar2000.org/components/view/foo_abx). Sixteen trials were applied for each of the comparisons between the original and 11-kHz high-cut sounds and between the original and 22-kHz high-cut sounds. Each trial provided four playback options (A, B, X, and Y). Sounds A and B were fixed, whereas sounds X and Y were randomly switched. The participants’ task was to choose which was true: “X is A, and Y is B” or “Y is A, and X is B.” They were allowed to repeat the sounds as many times as they wanted.

Finally, a self-administered auditory threshold test was conducted. Pure tones (starting from 10 kHz and increasing by 500 Hz) were present via the same playback devices used in the main experiment. Participants were asked to adjust a cursor to the highest frequency of tone that they could hear.

### Analysis

For MMN, only standard and deviant stimuli that occurred after at least two consecutive standard stimuli were analysed. The 4-ms delay between the stimulus trigger and sound onset was adjusted. The periods between 100 ms before and 500 ms after the sound onset were averaged separately for each type of white noise burst. The periods that contained a voltage exceeding ± 60 μV were excluded from ERP analysis. Due to technical failure, the data of three participants were excluded from the analysis. The data of the remaining 41 participants were submitted to reliability analysis using the ERP Reliability Analysis (ERA) Toolbox^28^. However, the dependency estimates did not reach the predefined level (i.e. 0.80). This situation often occurs when dealing with a stable signal with low between-person variance^29^. Therefore, the inclusion criterion described in the published ERP guidelines^26^ was applied instead: at a minimum, 150 trials were averaged for each deviant. According to this criterion, the data of 38 participants were retained, and those of 3 participants were removed.

The MMN response was assessed at the frontocentral electrode cluster (Fz, FC1, FC2, and Cz). The collapsed localiser approach^30^ was used to determine the latency range for measuring the MMN amplitude. Grand mean waveforms were calculated by collapsing across the two conditions. The peak was determined to lie between 110 and 230 ms on the deviant minus standard grand mean difference waveform. Then, the mean amplitude of the 40-ms period centred around the observed peak latency (i.e. 120–160 ms) was used to quantify the MMN response. A one-tailed *t* test was used to test the presence of the MMN response (i.e. the difference was smaller than zero, *p* < 0.05). In addition, a Bayesian paired sample *t* test was conducted by JASP^31^ to calculate the Bayes factor to evaluate evidence in favour of the null hypothesis (i.e. no MMN elicitation).

As an exploratory analysis, point-by-point *t* statistics were calculated on entire waveforms to examine possible differences between the deviant and standard ERP waveforms. The periods with significant differences were assessed against the critical value obtained by permutation tests corrected for Type I error inflation^32^. The calculation was done by an accessory function of the standardized low resolution brain electromagnetic tomography (sLORETA) software (http://www.uzh.ch/keyinst/loreta.htm). The number of randomizations was 10,000.

In the ABX test, 12 or more correct answers out of 16 trials were considered to be better than chance (*p* < 0.05, one-tailed). The mean and 95% confidence interval were calculated for the number of corrects in each condition. Pearson’s correlation coefficients were calculated between the MMN amplitude and the number of correct responses in the 11- and 22-kHz sound conditions, respectively, and between the MMN amplitude and the upper audible threshold. The violin plots were drawn using a free software HAD (https://norimune.net/had).

## Supplementary Information

Supplementary Information
