# Supplementary material for: High-frequency sound components of high-resolution audio are not detected in auditory sensory memory
Source: Sci Rep. 2020 Dec 10;10:21740. doi: 10.1038/s41598-020-78889-9 (PMC7730382; doi:10.1038/s41598-020-78889-9)
Supplement: Supplementary file 1 — Supplementary Information [file 41598_2020_78889_MOESM1_ESM.docx]

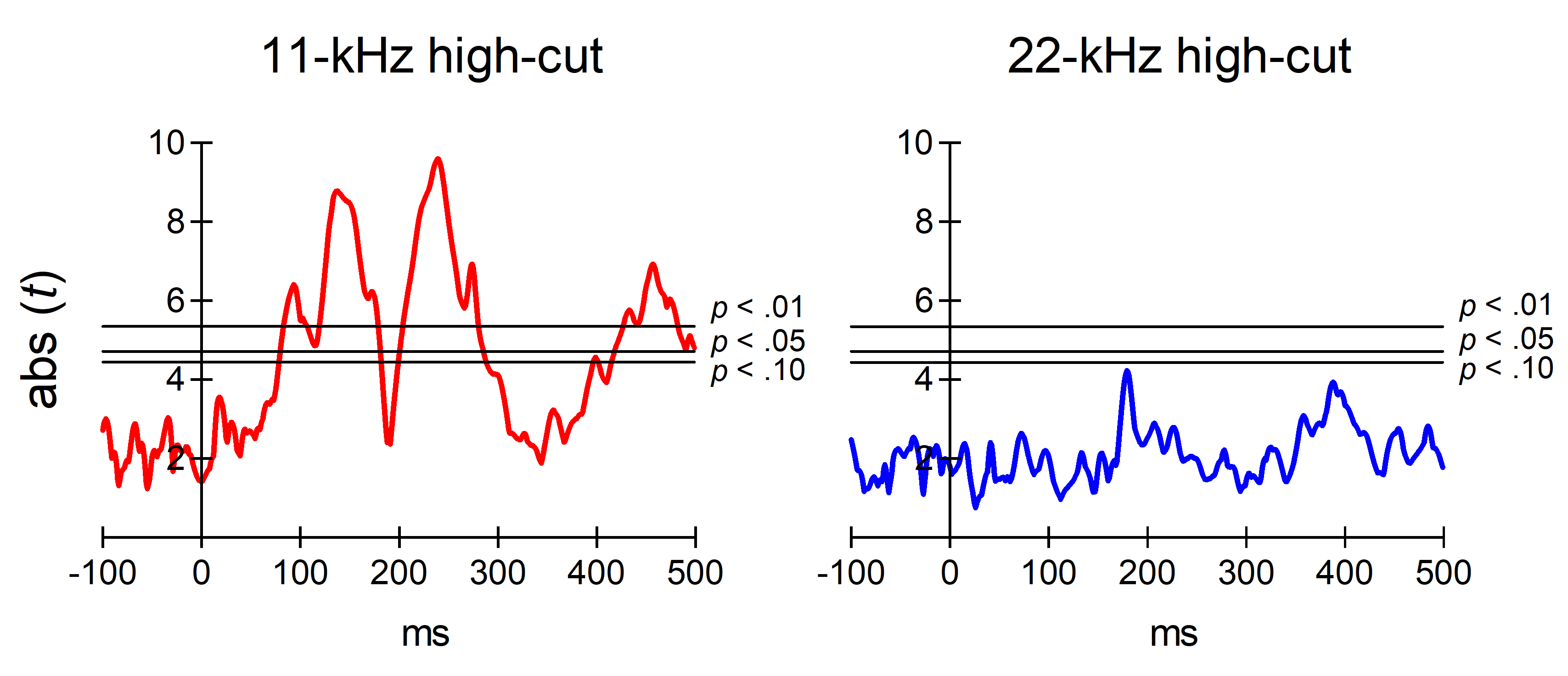


**Supplementary Figure S1. Significant differences between the deviant and standard ERP waveforms.** The maximum *t* value across all scalp electrodes at each time point is shown. The horizontal lines show the significance thresholds for the two-tailed *t* tests that were corrected for Type I error inflation.


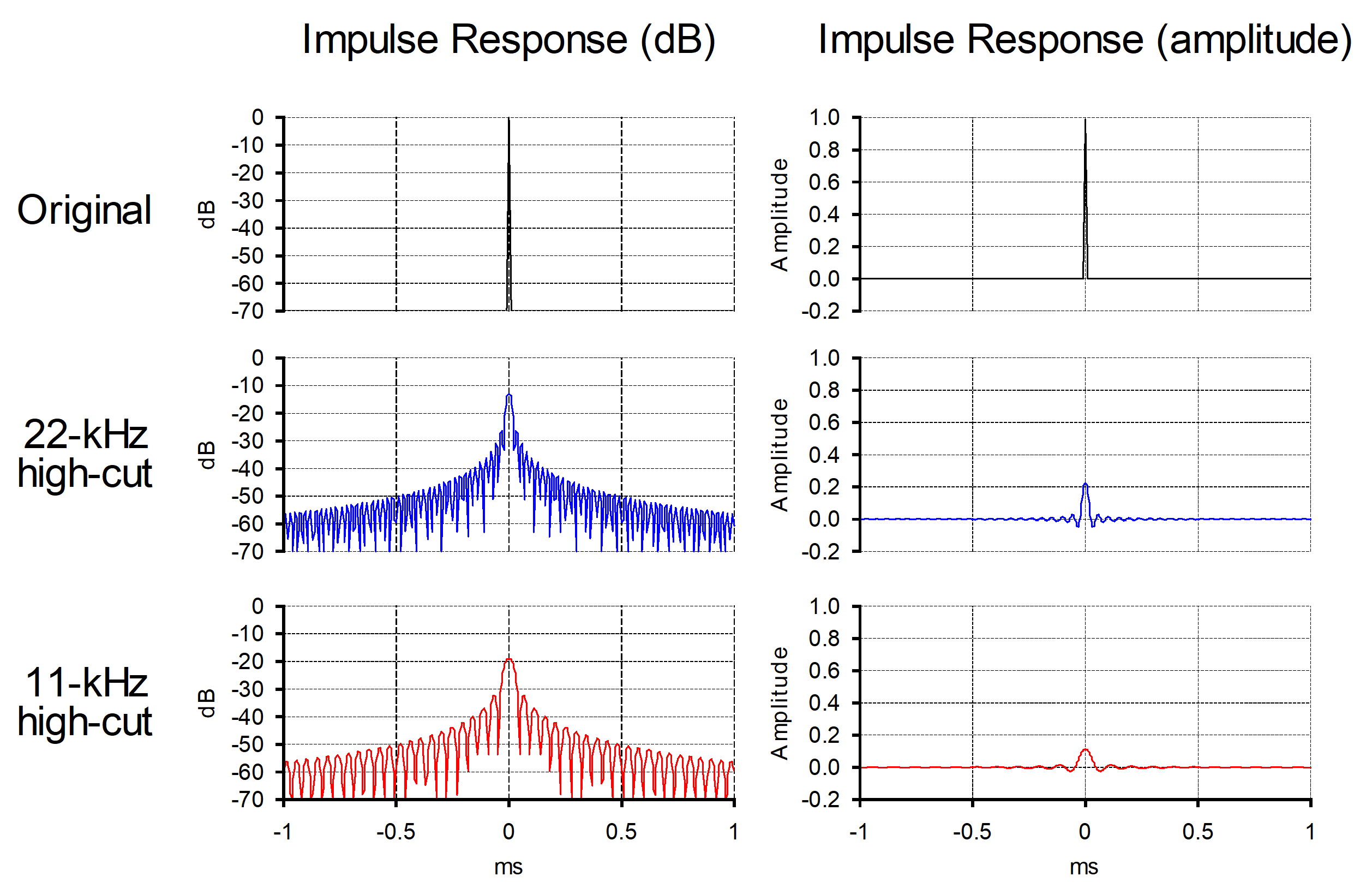


**Supplementary Figure S2. Impulse response characteristics of the anti-alias filters used in the present study.** No filter was applied to the original waveform.
